# Dissection of Seven Saline Tolerance Genes in a *Geng*/*Japonica* Population Suggests the Subspecies-Specific Differentiation at *RST1*^530A^ in Rice

**DOI:** 10.3390/cimb48101011

**Published:** 2026-10-01

**Authors:** Yuanhang Cheng, Haifeng Liu, Ruihua Zhang, Wenjie Feng, Wenbo Nie, Huadong Zhang, Zhaohui Chu

**Affiliations:** 1 State Key Laboratory of Hybrid Rice, Hubei Provincial Research Center for Basic Biological Sciences, College of Life Sciences, Wuhan University, Wuhan 430072, China; 2020302041124@whu.edu.cn (Y.C.); 2022302041083@whu.edu.cn (W.N.); 2025102040049@whu.edu.cn (H.Z.); 2 State Key Laboratory of Wheat Improvement, College of Agronomy, Shandong Agricultural University, Taian 271018, China; 3 Linyi Academy of Agricultural Science, Linyi 276012, China; zhangrhshuidao@163.com; 4 Jining Academy of Agricultural Sciences, Jining 272031, China; jnnky_fwj@126.com; 5 Ezhou Seed Technology Institute of Hubei Province, Ezhou 436043, China

**Keywords:** salt tolerance, *geng*/*japonica* rice, single nucleotide polymorphism (SNP), KASP, *RST1*

## Abstract

Improving salt tolerance (ST) is a critical objective for rice production in China’s Huang–Huai–Hai (3H) region, where soil salinity poses a persistent threat to crop productivity. Natural allelic variations in ST genes serve as vital genetic resources for addressing this challenge. In this study, we investigated nine single nucleotide polymorphisms (SNPs) associated with seven known ST genes across 215 *geng*/*japonica* rice varieties from this region, including *OsSTG5*, *SKC1*, *OsHKT1;1*, *OsHKT2;3*, *OsWRKY53*, *OsSTL1*, and *RST1*. These genes cover diverse functional categories—ion transport, transcriptional regulation, and QTL-based regulation—with known ST functions predominantly characterized in xian/indica rice, whereas their distribution and relevance in 3H *geng*/*japonica* germplasm remained unclear. Through KASP genotyping and haplotype analysis, these varieties were classified into 14 distinct haplotypes. ST level assessment of these SNPs revealed that *OsSTL1*, *OsHKT1;1*, *RST1* and *OsSKC1* variants significantly contribute to improved ST. Notably, *RST1*^530A^ was identified as a pivotal factor in enhancing ST (57.1% increase in relative shoot fresh weight, *p* < 0.01) and occurs in only 3.7% of the 3H *gene*/*japonica* varieties versus 93.6% in *xian*/*indica* accessions. Further physiological and molecular analyses demonstrated that the *RST1*^530A^ allele confers ST by attenuating repression of *OsAS1* (6.97-fold induction under salt stress vs. 4.55-fold for *RST1*^530G^), thereby increasing its expression under salt stress. Evolutionary analysis suggests that the *RST1*^530A^ allele in *geng*/*japonica* rice likely originated from *xian*/*indica* varieties through historical hybridization, involving introgression of a ≥68 Kb chromosomal segment (a minimum estimate based on flanking marker analysis). These varieties represent promising candidate donor parents for introducing *RST1*^530A^ into *geng*/*japonica* rice breeding programs, pending further agronomic evaluation under field conditions.

## 1. Introduction

The Huang–Huai–Hai (3H) rice region, a vital area for *geng*/*japonica* rice cultivation in China, typically refers to the agricultural zone formed by the alluvial plains where the Yellow, Huai, and Hai Rivers converge [1,2]. Rice yield in this region plays a crucial role in ensuring food security. The 3H rice region is bordered by the Bohai and Yellow Seas of China. Due to seawater intrusion, rising groundwater levels, and strong evaporation, the area of saline–alkali soil continues to expand, increasingly subjecting *geng*/*japonica* rice to salt stress during its growth [3,4,5]. This type of abiotic stress not only inhibits seed germination and seedling growth and development but also significantly reduces yield [6,7]. Therefore, breeding salt-tolerant *geng*/*japonica* rice varieties has become an urgent priority to ensure food security in this region [8]. Although many QTLs and genes have been identified to confer rice salt tolerance (ST), most of them remain at the theoretical level [9]. Only a few loci, like “Saltol”, have been reported as being applied for breeding salt-tolerant varieties [10]. Other loci that have been found to be related to ST need further functional tests in natural varieties, especially in those cultivated in saline–alkali areas, and varieties of the 3H rice region are appropriate for such assessment.

In the field of rice ST research, several key genes involved in ion balance, signal transduction, and transcriptional regulation have been identified from various materials [6,11]. However, most studies focus on *xian*/*indica* rice or model varieties, and the natural variation and breeding potential of these genes in *geng*/*japonica* rice—particularly local germplasm from the 3H region—remain unclear. For instance, *SKC1*, also referred to as *OsHKT1;5*, is a Na^+^ selective transporter of the HKT family in rice [12]. Expressed mainly in root stele and xylem parenchyma cells, it unloads Na^+^ from the xylem to reduce sodium buildup in aerial tissues and improve plant salt resistance [12,13,14]. It is the causal gene underlying the major ST QTL “Saltol” on rice chromosome 1 [10,15]. *OsWRKY53*, mapped by genome-wide association studies (GWAS), is a key negative regulator gene of ST, directly repressing the expression of the major salt-tolerant gene *SKC1* (*OsHKT1;5*) and *OsMKK10.2* [16]. The natural variation in *OsWRKY53* enhances ST predominantly in *xian*/*indica* rice varieties [16,17]. *STG5* was identified via a combined multi-omics and genetic mapping strategy and was found to regulate HKT family genes to contribute to ST [18]. The ST haplotype of STG5 is also predominantly enriched in *xian*/*indica* rice populations [17,18]. *RST1*, also referred to as *OsARF18*, negatively regulates ST in rice by directly repressing the transcription of *OsAS1*, a key gene in nitrogen metabolism [19,20]. Loss of *RST1* function (e.g., in the *rst1* mutant) completely abolishes this repression, improving nitrogen utilization and ultimately enhancing ST [19]. Two key single nucleotide polymorphisms (SNPs) cause the G530A and E611G amino acid substitutions in the *RST1* protein. Importantly, the G530A substitution does not represent a complete loss-of-function allele. Instead, it defines a hypomorphic variant that partially reduces the transcriptional repression activity of *RST1*. This partial reduction is sufficient to confer improved ST, as demonstrated by the enhanced performance of lines carrying the *RST1*^530A^ allele. The E611G substitution, located in the PB1 domain, may also contribute to modulating *RST1* activity, although its precise mechanism requires further investigation. Here and throughout this study, *RST1*^530A^ denotes the ST variant (alanine at position 530), whereas *RST1*^530G^ denotes the salt-sensitive variant (glycine at position 530). *OsHKT1;1* and *OsHKT2;3* are two HKT family genes whose expression and function are regulated by multiple upstream factors, including *HST1*, *OsWRKY53*, and *STG5* [16,19,21,22,23]. *OsSTL1* encodes a C2C2-type zinc finger protein on chromosome 4 and positively regulates ST in rice seedlings [24]. All these genes have not been investigated in specific cultivars in the 3H rice region in China, hindering the potential application of these ST genes for improving the ST of these varieties.

Natural variations cause different functional levels of gene alleles [25,26]. Some ST genes have been reported to be classified into haplotypes based on SNP differences, which provides us with a tool for functional evaluation and utilization of these genes [27,28]. Here, we collected nine SNPs distributed in seven ST-associated genes reported to be associated with ST, including *OsSTG5*^12S^, *SKC1*^140A^, *SKC1*^184H^, *OsHKT1;1*^94K^, *OsHKT2;3*^I77T^, *OsWRKY53*^173G^, *OsSTL1*^289S^, *RST1*^530A^ and *RST1*^E611G^ [12,16,17,18,20,22,23,24]. The aim of this study was to investigate the distribution and functional relevance of these SNPs in a population of 215 *geng*/*japonica* rice varieties in the 3H rice region in China, as well as to classify these varieties into distinct haplotypes based on the combination of the targeted SNPs. Through haplotype-based comparisons, we sought to evaluate the relative contribution of each SNP to ST in the *geng*/*japonica* rice population. In particular, we focused on validating the functional role of the *RST1*^530A^ allele using chromosome segment substitution lines (CSSLs), combined with targeted RT-qPCR and biochemical analyses, to elucidate the *RST1*-*OsAS1* regulatory cascade under salt stress. Furthermore, we aimed to trace the potential origin of favorable alleles identified in *geng*/*japonica* varieties through pedigree and linkage analysis. Our findings are expected to clarify the genetic architecture of ST in the 3H *geng*/*japonica* germplasm and to provide candidate alleles and donor lines for marker-assisted breeding aimed at enhancing ST in this important rice-producing region.

## 2. Materials and Methods

### 2.1. Rice Resources and Growth Conditions

The 215 Huang–Huai–Hai *geng*/*japonica* rice varieties were used in this study (Supplementary Table S1); among them, varieties 1–194 were from the collections as previously described [2], while varieties 195–215 were collected by Mr. Ruihua Zhang (Linyi Academy of Agricultural Science, Linyi, China). Prior to the experiment, seeds were dried at 37 °C for 3 d and then soaked in double-distilled water (ddH_2_O) for 3–4 d until germination. Germinated seeds were then sown in 96-well black plastic plant boxes containing ddH_2_O for 2 d. Subsequently, the water was replaced with Yoshida rice nutrient salts (Coolaber, Beijing, China). The nutrient solution was renewed every 3 d to ensure a stable nutrient supply. Rice seedlings were grown in a phytotron within the Plant Growth Breeding System (PGBS, Wuhan Greenfafa Institute of Novel Genechip R&D Co., Ltd., Wuhan, China) greenhouse with 12 h of light at 30 °C and 12 h of darkness at 26 °C, and the humidity maintained at 70% [17]. For each experiment, seeds from three independent batches (planted at different times) were used to ensure reproducibility.

### 2.2. SNP Genotyping Based on KASP and Cluster Analysis

Nine ST-associated nucleotide polymorphisms (SNPs) including *OsSTG5*^12S^, *SKC1*^140A^, *SKC1*^184H^, *OsHKT1;1*^94K^, *OsHKT2;3*^I77T^, *OsWRKY53*^173G^, *OsSTL1*^289S^, *RST1*^530A^ and *RST1*^E611G^ were used for genotyping analysis. Kompetitive Allele-Specific PCR (KASP) serves as a robust tool for genotyping SNPs within gene sequences [2,29]. In this assay, each SNP was designed with a group of KASP primers listed in Supplementary Table S2. One genotyping sample was a 5 μL mixture consisting of 50–100 ng genomic DNA, 0.05 μM each forward primer, and 0.15 μM reverse primer, added with 2.5 μL 2 × Master Mix for ASPCR V1 (HC Scientific, Chengdu, China). The PCR program was as follows: First, initial hot-start activation at 95 °C for 10 min; Then 10 touchdown cycles including 95 °C denaturation for 20 s and starting at 61 °C annealing/extension for 40 s, decreasing by 0.5 °C per cycle; Followed by 26 standard cycles, including 95 °C for 20 s and 56 °C for 40 s; Finally, fluorescence endpoint signals of FAM and HEX were scanned after amplification, and genotype clustering analysis was conducted using LGC SNP viewer software, version 5.1.1.27582 (LGC Genomics, Hoddesdon, UK) to distinguish homozygous reference alleles, homozygous variant alleles and heterozygous genotypes. Each variety was genotyped with two technical replicates per SNP; any discordant calls were resolved by a third replicate. The entire genotyping assay was independently repeated twice, yielding >99% concordance. Based on the genotyping of the nine SNPs, varieties with identical types at all SNPs were clustered into one haplotype. Then the genetic distance was calculated between different haplotypes based on the neighbor-joining method to create the phylogenetic tree [30].

### 2.3. Phenotype Assessment of ST

Phenotypic assessment of ST was performed following a previously described protocol [17] in which the concentration of 150 mM NaCl was validated for discriminating salt-tolerant and salt-sensitive rice varieties at the seedling stage and was therefore adopted in this study. In brief, two-week-old seedlings had the nutrient solution replaced with 150 mM NaCl to simulate a salt stress environment. The salt treatment lasted for 7 days, after which the NaCl-containing solution was replaced with normal nutrient solution for a 5-day recovery period (rewatering). Three phenotypic parameters were measured to evaluate ST: (1) Relative shoot fresh weight (rSFW)—Shoot fresh weight (SFW) was determined for each plant immediately after the recovery period. rSFW was calculated as the ratio of SFW in the treated group to the mean SFW of the corresponding control group. (2) Dead leaf rate (DLR)—The number of fully dead leaves (more than 50% chlorotic/necrotic area) was counted and expressed as a percentage of the total number of leaves per plant. (3) Relative maximum quantum efficiency of PSII (rFv/Fm)—Fv/Fm was measured before salt treatment (day 0) and immediately after the 7-day treatment using the PathoScreen plant pathological phenotype measurement system (PhenoVation, Wageningen, The Netherlands). rFv/Fm was calculated as the ratio of Fv/Fm after treatment to Fv/Fm before treatment. This parameter reflects the salt-induced inhibition of photosynthetic efficiency [31,32]. For the haplotype comparison assays and CSSL validation experiments, each genotype/treatment combination included at least three biological replicates, with each biological replicate consisting of 12 individual plants. The entire experiment was independently performed three times, and data from all three independent experiments were pooled for statistical analysis. In the case of rFv/Fm measurements, 6 plants per replicate were measured.

Calculation of contribution rates:

To evaluate the functional contribution of each ST-associated SNP to salt tolerance, the relative shoot fresh weight (rSFW) of the target haplotype (HapX) was compared with that of the reference haplotype (Hap1), which served as the baseline. The contribution rate for each SNP was calculated as the percentage increase in mean rSFW of the target haplotype relative to Hap1:

Contribution rate (%) = [(Mean rSFW of HapX − Mean rSFW of Hap1)/Mean rSFW of Hap1] × 100%

This metric represents the relative phenotypic improvement conferred by the additional ST-associated SNP(s) present in the target haplotype. Only haplotypes with at least three representative varieties were included in this analysis to ensure statistical robustness.

### 2.4. RNA Extraction and Quantitative Real-Time PCR

Total RNA was extracted from plant tissues using the RNApure Fast Plant Kit (CWBIO, Beijing, China) according to the manufacturer’s instructions. Briefly, tissue samples were ground in liquid nitrogen and lysed with RSL buffer. After gDNA removal, RNA was bound to the spin column, washed with RW1 and RW2 buffers, and eluted with RNase-free water. Reverse transcription was done for the synthesis of the first-strand cDNA using the HiFiScript gDNA Removal cDNA Synthesis Kit (CWBIO). Then, qRT-PCR was performed on the Archimed X4 Detection System (ROCGENE, Beijing, China) using the UltraSYBR Mixture kit (CWBIO). The amplification program was set as follows: Initial denaturation at 95 °C for 3 min, followed by 40 cycles of 95 °C for 10 s and 60 °C for 30 s. Melting curve analysis was conducted from 65 °C to 95 °C to verify amplification specificity. Each qRT-PCR reaction was run in three technical replicates. For each time point and genotype, three independent biological replicates were included (each biological replicate consisted of root tissue pooled from five individual plants). The entire qRT-PCR experiment, including salt treatment and RNA extraction, was independently performed twice, yielding consistent results; representative data from one independent experiment are presented in the main figures, with error bars representing SD from three biological replicates. Relative gene expression levels were calculated using the 2 − ∆∆Ct relative quantitative method with a reference gene for normalization [33]. The primers used for *RST1*, *OsAS1*, and *OsACTIN* are listed in Supplementary Table S3.

### 2.5. FBP-LUZ Reporter Assay for Transcription Repression Analysis

The fungal bioluminescence pathway (FBP) is an autonomous, caffeic acid-dependent luminescence system originating from fungi. The expression level of *LUZ* in the system determines the intensity of bioluminescence when caffeic acid is sufficient. FBP-LUZ was validated to function well in *Nicotiana benthamiana* leaves and has been used to evaluate the effect of transcription factors on target promoters [34]. Unlike conventional luciferase reporters, it enables continuous, substrate-free bioluminescence, making it a powerful tool for the non-invasive monitoring of gene expression in plants [35]. We constructed the FBP-LUZ system in three parts: (1) the reporter constructpAS1:LUZ, in which the 2 kb genomic region immediately upstream of the *OsAS1* start codon was inserted upstream of the *LUZ* reporter gene in the plasmid, pGreenII 0800; (2) the effector constructs *p35S:RST1*^530G^ and *p35S:RST1*^530A^, in which the coding sequence of *RST1*^530G^ and *RST1*^530A^ were cloned under the control of the CaMV 35S promoter in the pCAMBIA1300 vector; (3) the caffeic acid synthesis system (CHN), consisting of four genes (*HispsS*, *H3H*, *CPH* and *NPGA*) driven by *p35S* in the plasmid pCAMBIA1300, which provided the substrate (caffeic acid) for the FBP [36,37]. All constructs were verified by Sanger sequencing and individually transformed into *Agrobacterium tumefaciens* strain GV3101. For transient expression, 4-week-old *N. benthamiana* leaves were co-infiltrated with *Agrobacterium* cultures harboring the reporter, effector, and CHN constructs at a 1:1:1 ratio (OD_600_ = 0.6 each). After 48 h of infiltration, salt treatment was applied by spraying 100 mM NaCl solution evenly onto the leaf surface followed by a 3 h incubation. Then, luminescence signals were detected by the NightShade LB985 plant imaging system (Berthold Technologies, Bad Wildbad, Germany), which operates at a spectral range of 350–1050 nm. For each construct combination, at least four leaf disks from different leaves (biological replicates) were analyzed. The entire infiltration experiment was independently repeated three times, and luciferase deviation (relative luminescence units normalized to the pAS1:LUZ-only control) was quantified using the instrument’s software. JAMYB + pAGO18:LUZ, a validated positive control for transcriptional activation [35], was included to ensure that the system was functioning properly.

### 2.6. Statistical Method for Data Analysis

All statistical analyses were performed using SPSS Statistics (version 26.0, IBM Corp., Armonk, NY, USA) and GraphPad Prism (version 9.0, GraphPad Software, San Diego, CA, USA). Data were first tested for the normality (Shapiro–Wilk test) and homogeneity of variances (Levene’s test) to ensure compliance with parametric test assumptions.

For comparisons between two independent groups, the independent-samples *t*-test was applied. For comparisons involving three or more groups, one-way analysis of variance (one-way ANOVA) was performed, followed by Tukey’s honestly significant difference (HSD) post hoc test for multiple pairwise comparisons when the overall ANOVA was significant (*p* < 0.05). In cases where a single control group was compared to multiple treatment groups, Dunnett’s post hoc test was used instead.

For experiments with two independent variables (e.g., genotype × treatment), two-way ANOVA was performed, followed by Sidak’s multiple comparisons test.

All data are presented as mean ± standard deviation (SD) from at least three biological replicates. Data from three independent experiments were pooled for analysis unless otherwise noted, and the experimental replicate was treated as a random factor in the ANOVA model to account for batch-to-batch variation. A statistically significant difference was defined as *p* < 0.05. Significance levels are indicated in the figures as: *p* < 0.05, *p* < 0.01, *p* < 0.001; n.s., not significant.

## 3. Results

### 3.1. Distribution of ST Genes in a Huang–Huai–Hai Geng/Japonica Rice Population

**Figure 1:**
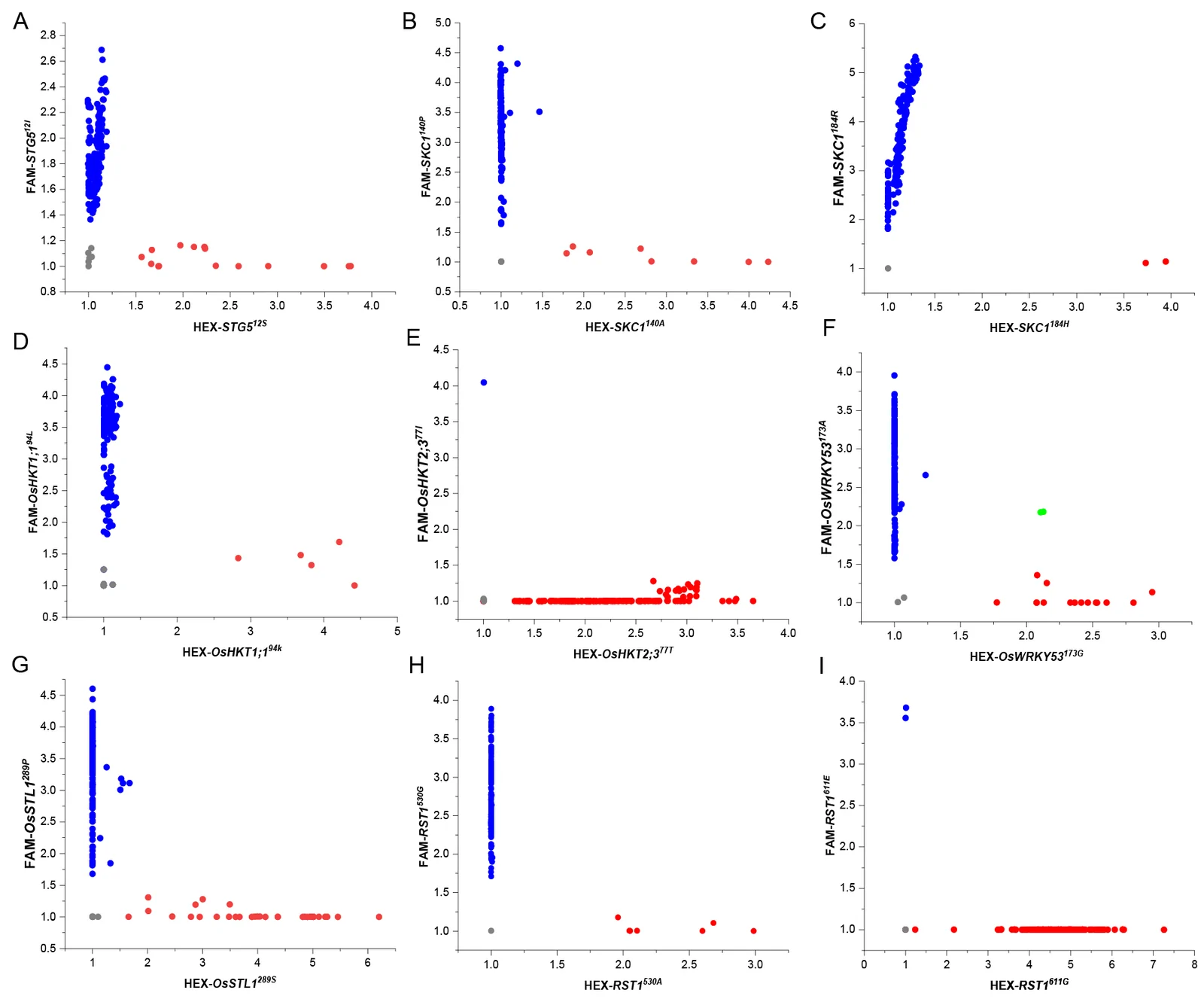
The genotyping maps of nine SNP loci based on KASP. (**A**–**I**) indicate the SNPs conforming to *OsSTG5*^12S^ (**A**), *SKC1*^140A^ (**B**), *SKC1*^184H^ (**C**), *OsHKT1;1*^94K^ (**D**), *OsHKT2;3*^77T^ (**E**), *OsWRKY53*^173G^ (**F**), *OsSTL1*^289S^ (**G**), *RST1*^530A^ (**H**) and *RST1*^611G^ (**I**), respectively. The fluorescence value on the horizontal axis represents HEX, and the fluorescence value on the vertical axis represents FAM. The orange dot indicates that a certain variety is classified as salt-tolerant at this SNP locus, the blue dot indicates that a certain variety is classified as salt-sensitive at this SNP locus, and the gray dot indicates a blank control or that this SNP of a certain variety has not been detected.

To investigate the distribution of ST genes in the *geng*/*japonica* rice population, we selected seven cloned ST genes, including *STG5*, *SKC1*, *OsHKT1;1*, *OsHKT2;3*, *OsWRKY53*, *OsSTL1* and *RST1*, and investigated their functional single nucleotide polymorphism (SNP) loci in 215 *geng*/*japonica* rice varieties belonging to the 3H *geng*/*japonica* rice region in China. The functional SNP loci included *OsSTG5*^12S^, *SKC1*^140A^, *SKC1*^184H^, *OsHKT1;1*^94K^, *OsHKT2;3*^77T^, *OsWRKY53*^173G^, *OsSTL1*^289S^, *RST1*^530A^ and *RST1*^611G^ according to previous reports [17]. The number in the superscript at each site represents the position of the amino acid, and the amino acid typing before and after the number corresponds to the salt-sensitive and salt-tolerant types, respectively. We used KASP to realize the typing of the above nine SNP loci. The genotyping maps of the nine SNP loci are shown in Figure 1A–I, which showed the availability of these primers, and certain genotypes of each SNP in each variety are listed in Supplementary Table S1.

**Figure 2:**
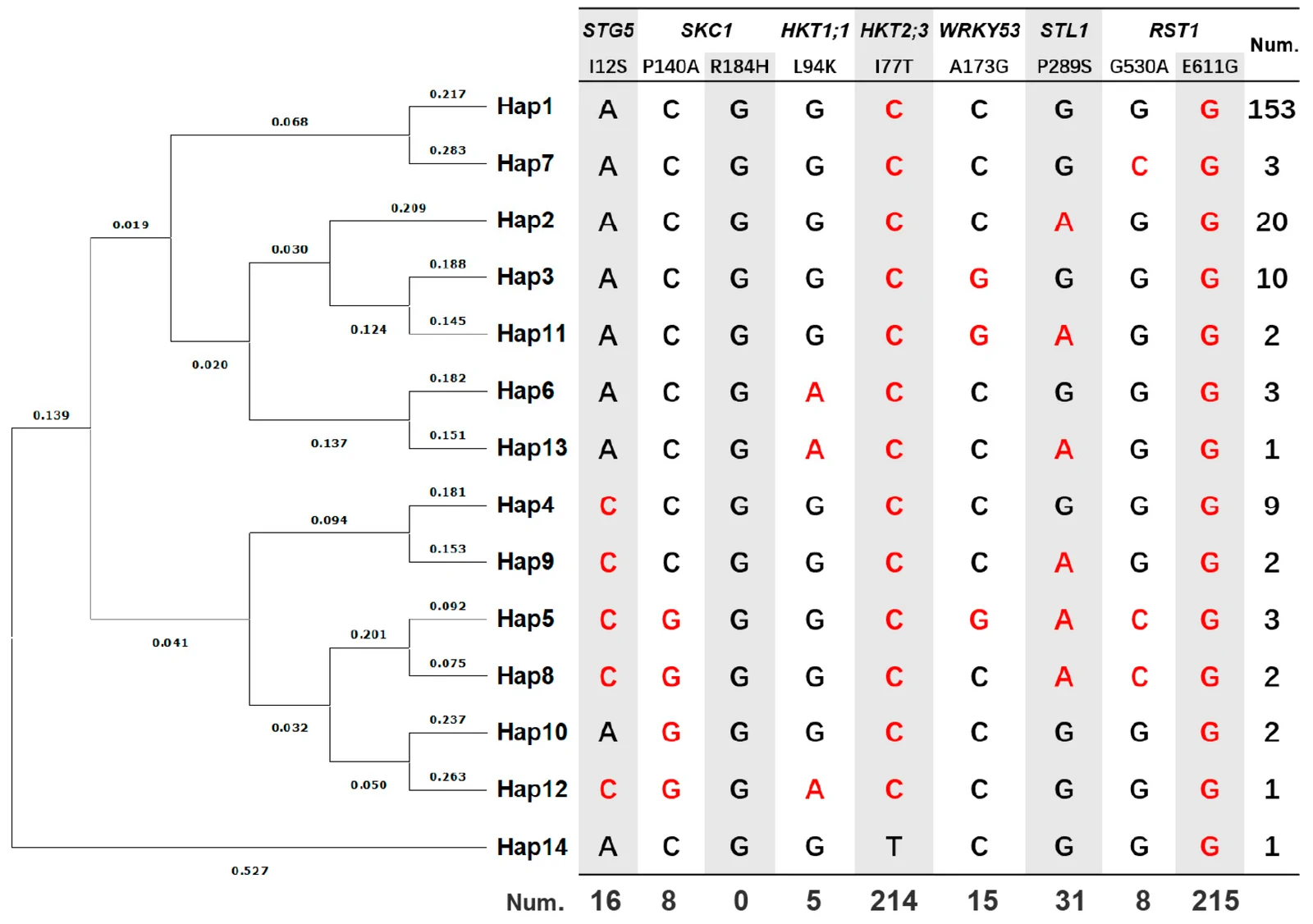
The figure shows the distribution of ST-associated SNP genotypes in the *geng*/*japonica* rice population of the Huang–Huai–Hai rice region. The left evolutionary branch represents the genetic distance between each haplotype, and the right side shows the specific genotypes of each SNP locus and the number of varieties included for each haplotype. The salt-tolerance SNP genotypes were marked in red.

Then, we compared the typing of various SNP loci in different varieties and found that 71.2% (153/215) of *geng*/*japonica* rice varieties had the same typing at each locus (Figure 2). The 153 varieties were classified as the major haplotype group of Hap1, and only two out of the nine SNP loci, including *OsHKT2;3*^77T^ and *RST1*^611G^, were ST type in the Hap1 varieties. The other haplotype groups (Haps) were listed from Hap2 to Hap14 in the order of the number of varieties included. The genetic distances between different Haps were calculated and shown in Figure 2. Correspondingly, *OsHKT2;3*^77T^ and *RST1*^611G^ were shared with up to 99.53% and 100%, respectively, in the investigated varieties. ST types at other SNP loci were much fewer than *OsHKT2;3*^77T^ and *RST1*^611G^; listed from high to low order, they were *OsSTL1*^289S^ (14.42%, 31/215), *STG5*^12S^ (7.44%, 16/215), *OsWRKY53*^173G^ (6.98%, 15/215), *SKC1*^140A^ (3.72%, 8/215), *RST1*^530A^ (3.72%, 8/215), and *OsHKT1;1*^94K^ (2.32%, 5/215), and no variety had the ST type, *SKC1*^184H^.

### 3.2. Functional Assessment of Six ST Genes in the Geng/Japonica Rice Population

**Figure 3:**
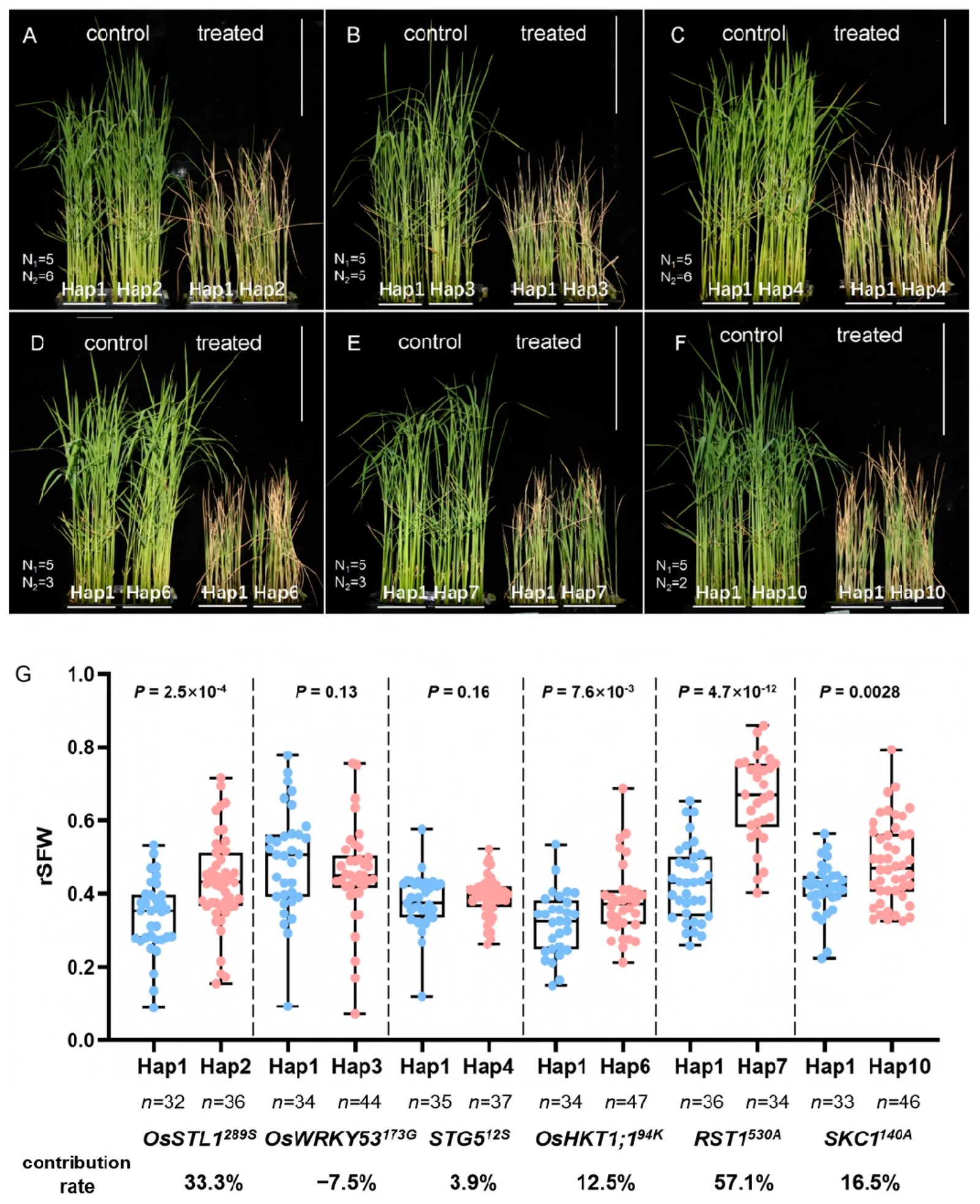
Functional assessment of ST-related genes in the *geng*/*japonica* rice population. (**A**–**F**) Images for evaluation of relative ST for Haps containing *OsSTL1*^289S^, *OsWRKY53*^173G^, *OsSTG5*^12S^, *OsHKT1;1*^94K^, *RST1*^530A^, and *SKC1*^140A^. Seedlings in the control group grew normally in the nutrient solution, while those in the treated group were grown in the nutrient solution supplemented with 150 mM NaCl for 7 days and then rewatered for 5 days. The scale bar represents 10 cm. N_1_ and N_2_ indicate the number of Hap1 and compare Haps used in the experiment, respectively. (**G**) Statistical analysis of ST level assessment of Haps compared with Hap1. *n* represents the number of individual plants statistically analyzed for the corresponding haplotype. The statistical method was a two-tailed *t*-test for independent samples.

To evaluate the contribution of each SNP locus to ST, we compared ST levels between specific haplotype groups according to the method previously described [17]. Because most varieties belonged to Hap1, which contains three conserved SNPs, we selected several varieties of Hap1 as a control and compared ST between the control and varieties representing each of the other haplotypes (Hap2–4, 6, 7, and 10). As mentioned above, Hap1 had just two ST-type SNP loci, while Hap2 carried three ST-type SNP loci, with an additional locus of *OsSTL1*^289S^. Therefore, we compared ST between Hap2 and Hap1 to evaluate the function of *OsSTL1*^289S^. Similarly, we compared Hap1 with Hap3, Hap4, Hap6, Hap7, and Hap10, respectively, to assess the ST function of *OsWRKY53*^173G^, *OsSTG5*^12S^, *OsHKT1;1*^94K^, *RST1*^530A^, and *SKC1*^140A^. Selected varieties were treated with 150 mM NaCl for seven days, then rewatered for an additional five days. Then, relative shoot fresh weight (rSFW) was measured as an ST index. The results showed that the genotypes of *OsSTL1*^289S^, *OsHKT1;1*^94K^, *RST1*^530A^, and *SKC1*^140A^ significantly contributed to ST improvement, with contribution rates of 33.3%, 12.5%, 57.1%, and 16.50%, respectively (Figure 3A,D–G). The number of varieties used for each haplotype comparison is indicated in Figure 3G (as *n* values), with the sample sizes balanced across compared groups.

### 3.3. RST1^530A^ Impaired the Repression of OsAS1 and Genetically Associated with ST

**Figure 4:**
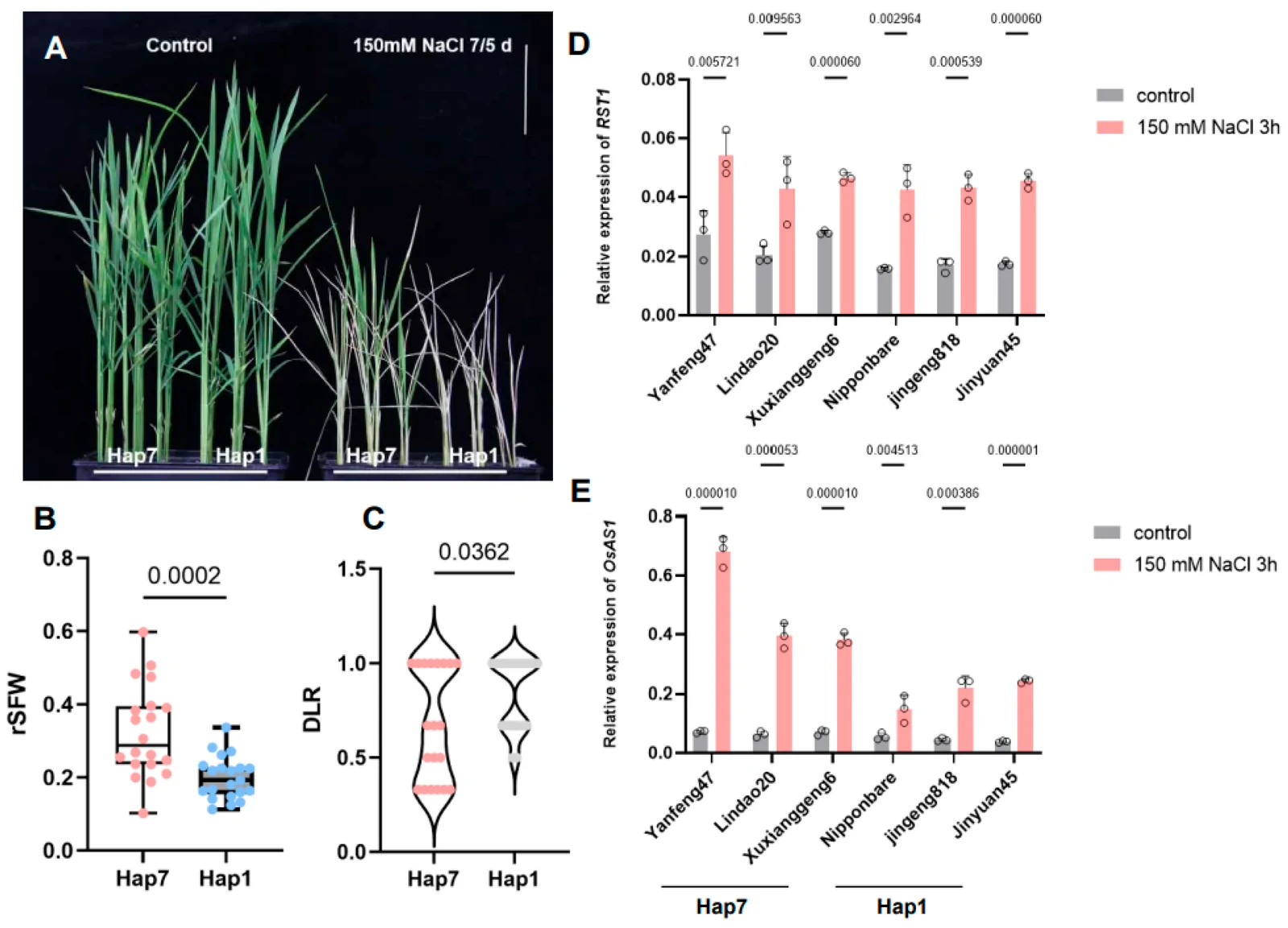
Assessment of the relative ST and transcription level of *OsAS1* between Hap7 and Hap1. (**A**) The phenotype of Hap7 and Hap1 in the control group and salt-treated group. The scale bar represents 5 cm. (**B**,**C**) The comparison of rSFW (**B**) and DLR (**C**) between Hap7 and Hap1. (**D**,**E**) The relative expression level of *RST1* (**D**) and *OsAS1* (**E**) between varieties in Hap7 and Hap1 in the control group and salt-treated group. The statistical method was a two-tailed *t*-test for independent samples.

In the ST comparison experiment, we noticed that Hap7 showed the highest ST level compared with Hap1, indicating that *RST1*^530A^ plays a pivotal role in improving ST. The result was consistent with our previous assessment of a *xian*/*indica* rice population [17]. Previous studies have shown that RST1 is a negative regulator of ST, reducing rice ST by repressing the target gene, *OsAS1* [19]. OsAS1 participates in the primary assimilation of NH^4+^ and catalyzes the synthesis of asparagine in roots. The loss of function of *RST1* results in the upregulation of *OsAS1*, which enhances nitrogen utilization efficiency by promoting asparagine synthesis and mitigates excessive NH_4_^+^ accumulation, thereby improving plant ST [19,20]. It is important to note that the *rst1* mutant described above represents a complete loss-of-function allele. In contrast, the RST1^530A^ variant examined in this study is a naturally occurring hypomorphic allele that partially reduces—but does not abolish—RST1’s repressive activity. This distinction is critical for interpreting the subsequent CSSL and FBP-LUZ results, which demonstrate that even a partial reduction in RST1 function is sufficient to confer enhanced salt tolerance. We hypothesized that the variation in *RST1*^G530A^ caused a nonsynonymous mutation might affect the *RST1-OsAS1* regulation axis and impact rice ST. To verify this hypothesis, three varieties of Hap7 (*Yanfeng47*, *Lindao20*, and *Xuxiangjing6*) and three varieties of Hap1 (*Nipponbare*, *Jingjing818*, and *Jinyuan45*) were used for a salt-tolerant comparison. Simultaneously, we detected the expression of *RST1* and *OsAS1* of these varieties before and after salt stress in root tissue. The results showed Hap7 had higher rSFW and lower DLR than Hap1 (Figure 4A–C). qRT-PCR showed that both *RST1* and *OsAS1* were upregulated after 3 h of treatment with 150 mM NaCl. The average fold changes of *RST1* were 1.94 in Hap7 and 2.60 in Hap1, and the average fold changes of *OsAS1* were 6.97 in Hap7 and 4.55 in Hap1 (Figure 4D,E). These results indicated that *RST1*^530A^ leads to slightly more impaired repression of *OsAS1* expression than *RST1*^530G^ under salt stress, which confers stronger ST in Hap7 than Hap1. While these qRT-PCR data indicate an association between *RST1* transcript levels and *OsAS1* expression, the FBP-LUZ assay (see Section 3.5) was used to determine whether the G530A substitution itself alters RST1’s repressive activity independent of transcript abundance.

### 3.4. An Introgression Line of RST1^530G^ Impaired the ST and Increased the Repression of Salt-Induced OsAS1 Expression

**Figure 5:**
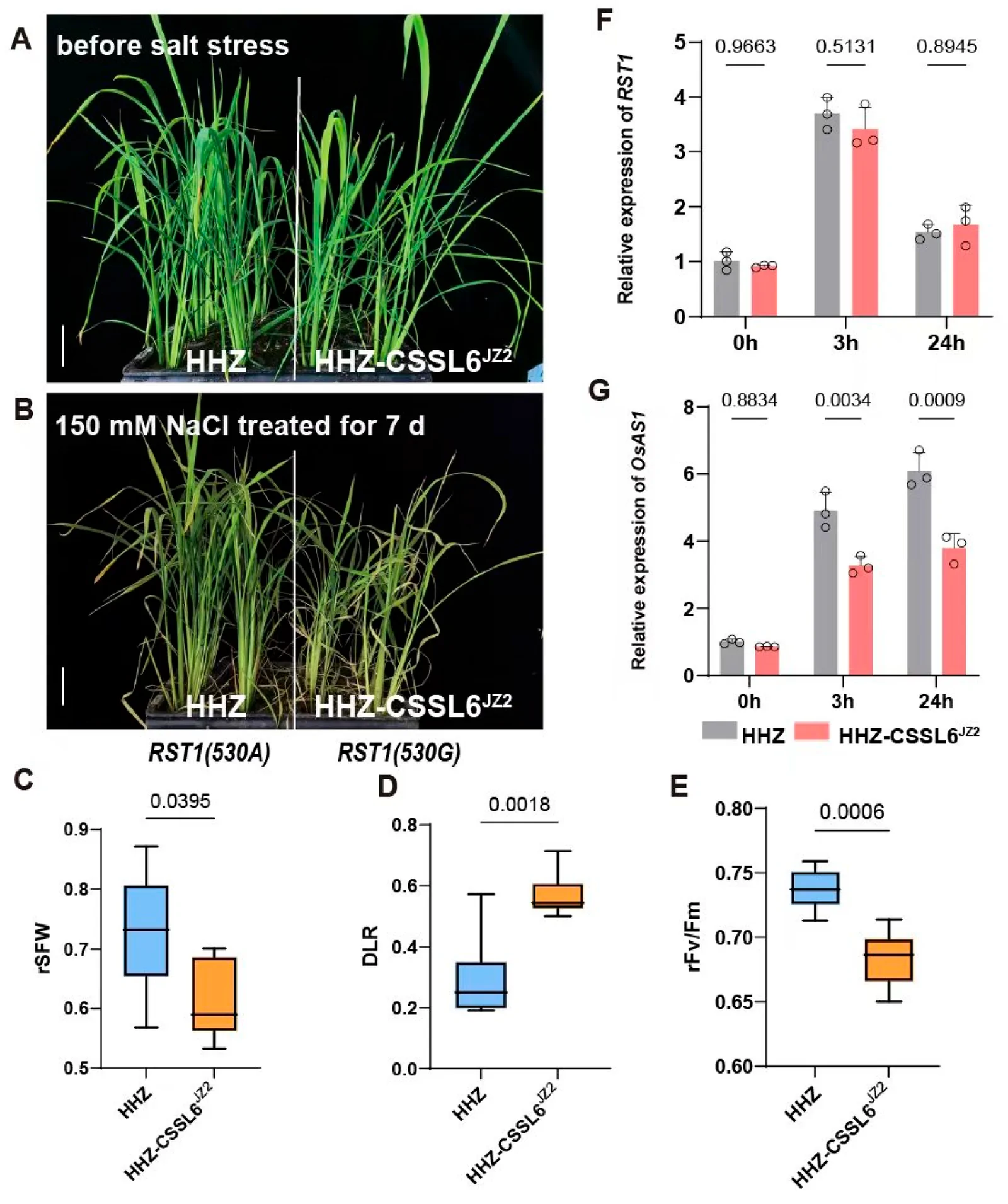
A chromosome segment substitution line of *RST1*^530G^ impaired the ST as well as increased the repression of salt-induced *OsAS1* expression. (**A**,**B**) The growth performance of HHZ and HHZ-CSSL6^JZ2^ before treatment with salt stress (**A**) and after being treated with 150 mM NaCl for 7 d (**B**). The scale bar represents 5 cm. (**C**–**E**) The rSFW (**C**), DLR (**D**) and rFv/Fm comparison between HHZ and HHZ-CSSL6^JZ2^. (**F**,**G**) The relative expression of *RST1* (**F**) and *OsAS1* (**G**) was quantified for HHZ and HHZ-CSSL6^JZ2^, respectively, at 0 h, 3 h, and 24 h post-salt treatment. The statistical method was a two-tailed independent-samples *t*-test for Figure 5C–G.

To further validate the effect of *RST1*^530A^ on ST, forward genetic assessment was performed. Huanghuazhan (HHZ) was a conventional planting *xian*/*indica* rice variety carrying *RST1*^530A^, while Jizi 2 (JZ2) was a conventional *geng*/*japonica* rice variety carrying *RST1*^530G^. Using HHZ as the recurrent parent and JZ2 as the donor introgression parent, lines of HHZ genetic background with a chromosome segment substitution line (CSSL) of JZ2 ranging from Chr6_27105342 to Chr6_29615495 were generated and named HHZ^CSSL6-JZ2^. We planted HHZ and HHZ^CSSL6-JZ2^ lines in Yoshida solution and compared ST between them (Figure 5A,B). The results showed that HHZ^CSSL6-JZ2^ had significantly lower rSFW and rFv/Fm, as well as a higher dead leaf rate (DLR) than HHZ (Figure 5C–E). Then we compared the transcription level of *RST1* and its target gene *OsAS1* in the roots of HHZ and HHZ^CSSL6-JZ2^, respectively, at 0 h, 3 h, and 24 h post-treatment with 150 mM NaCl. The results indicated that the expressions of *RST1* and *OsAS1* were both induced by salt stress as reported before (Figure 5F,G). Notably, the expression level of *RST1* was comparable between HHZ and HHZ-CSSL6-JZ2 before and after salt stress (Figure 5F), indicating that the observed difference in *OsAS1* expression between the two lines is not attributable to altered *RST1* transcript abundance. Instead, because HHZ carries *RST1*^530A^ and HHZ-CSSL6-JZ2 carries *RST1*^530G^ on an otherwise identical genetic background, the significantly higher OsAS1 expression in HHZ under salt stress (Figure 5G) reflects a functional difference in the repressive activity of the *RST1* protein itself. However, given that the CSSL introgressed segment spans approximately 2.5 Mb and may contain additional genes that could contribute to the salt-tolerance phenotype, these results are interpreted as supporting *RST1* as a major functional contributor within this introgressed tract, rather than as definitive proof of its exclusive role (see Section 4).

### 3.5. FBP-LUZ-Based Assay Revealed That RST1^530A^ Impaired the Repression of Salt-Induced OsAS1 Expression

**Figure 6:**
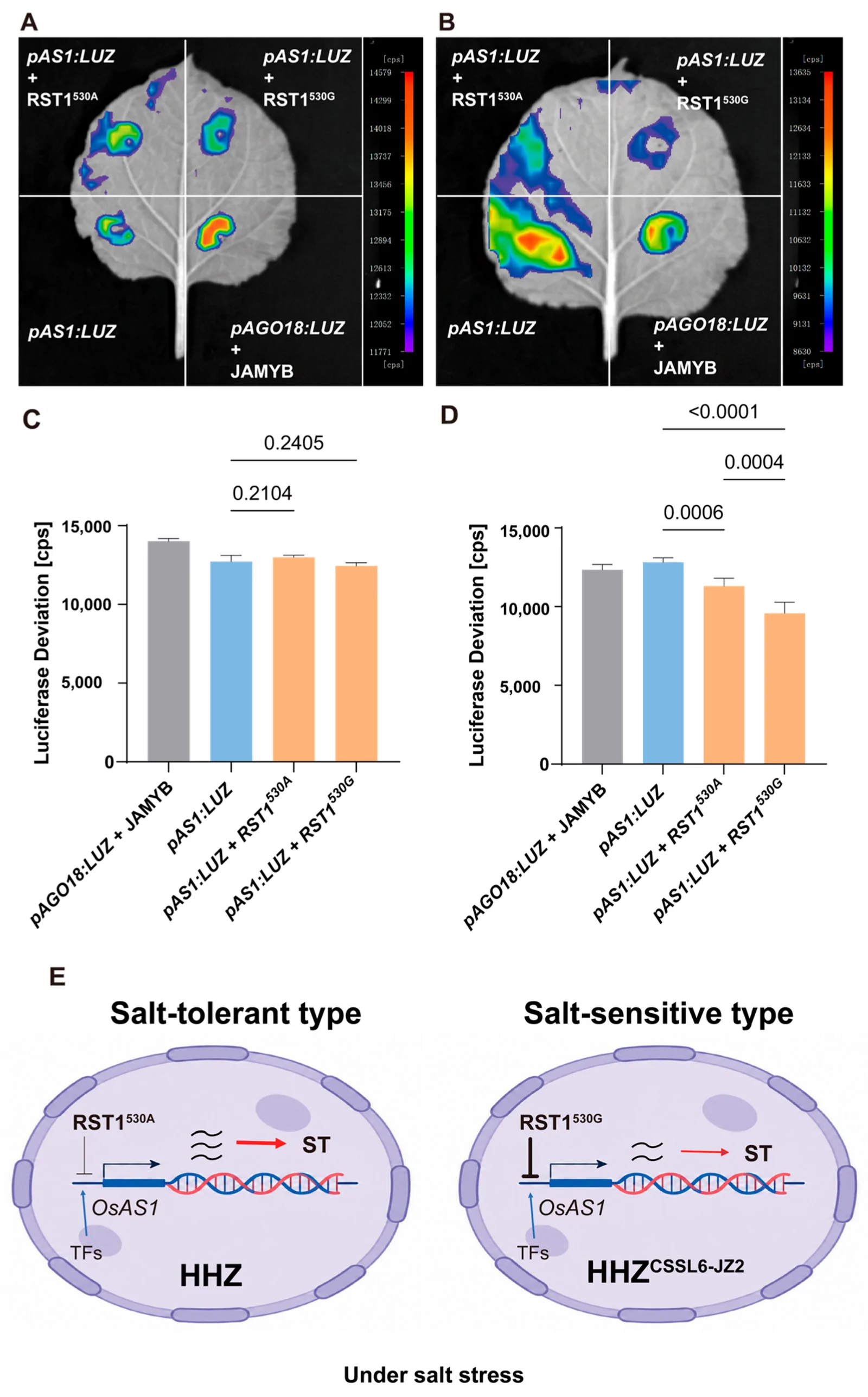
The FBP-LUZ-based assay revealed that *RST1*^530A^ impaired the repression of salt-induced *OsAS1* expression. (**A**,**B**) Luciferase detection in *pAS1:LUZ*, *RST1*^530G^ + *pAS1:LUZ*, *RST1*^530A^ + *pAS1:LUZ,* and JAMYB + *pAGO18:LUZ* without (**A**) and with (**B**) salt treatment. (**C**,**D**) Luciferase deviation of *pAS1:LUZ*, *RST1*^530G^ + *pAS1:LUZ*, *RST1*^530A^ + *pAS1:LUZ,* and JAMYB + *pAGO18:LUZ* without (**C**) and with (**D**) salt treatment. (n ≥ 4). The statistical method used was one-way ANOVA in Figure 6C,D. (**E**) Schematic model of *RST1*-mediated salt tolerance regulation in resistant versus sensitive genotypes.

To directly reflect the influence of different Haps of *RST1* on the promoter of *OsAS1*, we performed an FBP-LUZ (Fungal Bioluminescence Pathway-based *LUZ* reporter system) assay. The FBP-LUZ assay was recently developed for detecting transcription factor (TF) activity, which requires no exogenous substrates and possesses the advantages of high sensitivity, low background, and stable signals [35]. The genomic region spanning the 2 Kb sequence upstream of the start codon of *OsAS1* was used as the promoter and inserted upstream of the reporter gene *LUZ*, designated as *pAS1:LUZ*. It was co-transiently expressed with *RST1*^530G^ or *RST1*^530A^ protein, respectively. The leaves of *N. benthamiana* were divided into four parts; each part was co-expressed using the CHN expression system. As demonstrated in Figure 6A,B, compared to *pAS1:LUZ* and a control of JAMYB + *pAGO18:LUZ* [35], there was no change for the co-expressed groups of RST1^530G^ + *pAS1:LUZ* and RST1^530A^ + *pAS1:LUZ* without salt treatment. Under stress, the luciferase activity of both transient groups, RST1^530G^ + *pAS1:LUZ* and RST1^530A^ + *pAS1:LUZ*, was significantly lower than that of *pAS1:LUZ*, and that of RST1^530A^ + *pAS1:LUZ* was significantly higher than *RST1*^530G^ + *pAS1:LUZ* (Figure 6C,D). These results demonstrated that both Haps of *RST1* repressed the promoter activity of *OsAS1*, and *RST1*^530A^ exhibited weaker inhibition than *RST1*^530G^ (Figure 6E).

### 3.6. Evolutionary Analysis Reveals RST1^530A^ of Geng/Japonica Rice Varieties Originated from Specific Xian/Indica Ones

**Figure 7:**
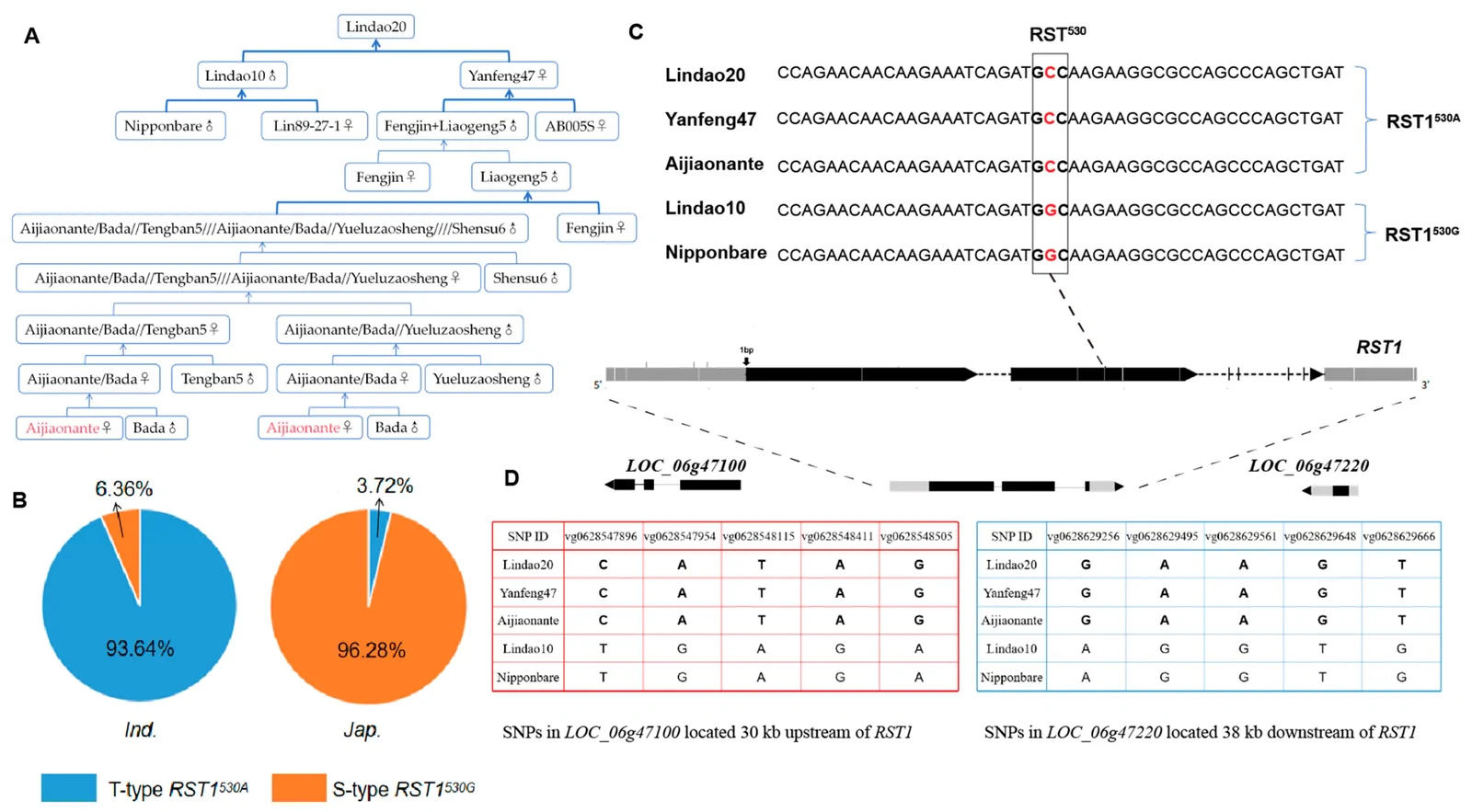
*RST1*^530A^ in *geng*/*japonica* rice *Lindao20* originated from the *xian*/*indica* variety *Aijiaonante*. (**A**) Pedigree diagram of *Lindao20*. Blue arrows point to progeny. Bold blue braces and arrows indicate varieties derived from crossbreeding, while non-bolding markers represent direct offspring from a single cross. (**B**) Distribution of *RST1*^530G^ and *RST1*^530A^ in *xian*/*indica* and *geng*/*japonica* rice populations. (**C**) Sequence alignment of *RST1*^G530A^ and adjacent region between *Lindao20*, *Yanfeng47*, *Aijiaonante*, *Lindao10* and *Nipponbare*. (**D**) SNPs of *LOC_06g47100* and *LOC_06g47220* in *Lindao20*, *Yanfeng47*, *Aijiaonante*, *Lindao10*, and *Nipponbare*. SNP IDs are available at https://ricerc.sicau.edu.cn/.

Only three varieties carrying *RST1^530A^* were identified in Hap7 (Figure 2), including *Lindao20* and its parent line *Yanfeng47* (Figure 7A). We previously revealed that the proportion of *RST1*^530A^ in a *xian*/*indica* rice population reached 93.64% as reported [17], while the proportion of *RST1*^530A^ was only 3.7% in the 3H *geng*/*japonica* rice population (Figure 7B). Thus, we speculated that *RST1*^530A^ in a few *geng*/*japonica* rice varieties may originate from *xian*/*indica* rice varieties during the process of subspecies hybridization breeding.

To verify this assumption, we investigated the sequence of the *RST1* gene in *Lindao20* and *Yanfeng47*. *Lindao20* was a progeny selected from a cross of *Lindao10* (♂) with *Yanfeng47* (♀), which originated from the donor parent *xian*/*indica* rice variety *Aijiaonante* by the Yinan County Rice Research Institute in China (Figure 7A). We found that both *Lindao20* and *Yanfeng47* had six variations compared to *Nipponbare* in the *RST1* coding region, including five SNPs at 565 bp, 1612 bp, 1743 bp (*RST1*^G530A^), 1986 bp, and 2102 bp, as well as one “CCG” INDEL at 744 bp (Figure 7C, Supplementary Figure S1). While *Lindao10* had an identical sequence of *RST1* to *Nipponbare*, this indicated that the *RST1* gene in *Lindao20* originated from *Yanfeng47*. *Aijiaonante* was an early *xian*/*indica* rice variety used as one of the donor lines on the in hierarchical diagram of *Lindao20* (Figure 7A). We found that *Aijiaonante* had the same sequence of *RST1* as *Lindao20* (Figure 7C, Supplementary Figure S1), suggesting that *RST1*^530A^ in *Lindao20* originated from *xian*/*indica Aijiaonante*. Additionally, *LOC_06g47100* and *LOC_06g47220* are two flanking genes, which are located 30 Kb upstream and 38 Kb downstream of *RST1*, respectively. We cloned each gene from all four rice varieties (*Lindao20*, *Yanfeng47*, *Aijiaonante*, and *Lindao10*) and aligned them with the reference sequence of *Nipponbare*. Five SNPs for each gene were mined, and they are classified into two Haps, with *Lindao20*, *Yanfeng47* and *Aijiaonante* grouped together while *Lindao10* and *Nipponbare* grouped as the other Haps (Figure 7C,D). These results suggest that a chromosomal segment of at least 68 Kb carrying the *RST1*^530A^ allele was transferred from the *xian*/*indica* rice variety *Aijiaonante* into the *geng*/*japonica* rice varieties *Lindao20* and *Yanfeng47*.

## 4. Discussion

### 4.1. Subspecies-Specific Differentiation of ST Allele Frequencies

Previously, alleles associated with ST had been found largely differentiated between *xian*/*indica* and *geng*/*japonica* rice populations [38,39,40,41]. However, the specific ST genes were not well-documented. A striking finding emerging from the comparison between our previous *xian*/*indica* study [17] and the present *geng*/*japonica* survey is the dramatic difference in the frequencies of ST alleles. Among the nine investigated SNPs, seven showed a markedly lower frequency in the *geng*/*japonica* population (215 Huang–Huai–Hai varieties) than in the *xian*/*indica* population (550 accessions). Specifically, *OsSTL1*^289S^ is fixed in *xian*/*indica* (100%) but accounts for only 14.4% in *geng*/*japonica*; *OsWRKY53*^173G^ drops from 92.6% to 7.0%; *RST1*^530A^ declines from 93.6% to 3.7%; *SKC1*^140A^ declines from 79.3% to 3.7%; and *SKC1*^184H^ is completely absent in *geng*/*japonica*. Only two *ST*-alleles, *OsHKT2;3^77T^* and *RST1*^611G^, shared similarly high frequencies in both subspecies (88.6–99.5% and 93.8–100%, respectively). This pattern raises the possibility that these alleles are ancient components of the ST machinery that may have already been present before subspecies divergence, although formal evolutionary evidence (e.g., population genetic tests of selection or divergence time estimation) would be required to confirm this hypothesis. However, the observed frequency contrasts alone do not constitute formal evidence of selection; they may also reflect demographic history, genetic drift, or differential introgression. Distinguishing these possibilities would require formal population genetic tests (e.g., F-ST outlier analysis, XP-CLR, or extended haplotype homozygosity) that are beyond the scope of this study.

What might explain this pronounced subspecific divergence? Several hypotheses can be proposed. First, differential selection pressures during domestication and breeding likely played a major role. *Xian*/*indica* rice is predominantly cultivated in southern China and Southeast Asia, where hot, humid climates often coincide with naturally or anthropogenically salinized soils (e.g., coastal areas, irrigation-induced salinity) [39,40,41]. In such environments, alleles that confer even modest ST would be strongly favored. In contrast, the 3H region, the key *geng*/*japonica* production area in northern China, experiences lower temperatures and historically less acute soil salinity, and breeding efforts have focused primarily on yield and cold tolerance, with only indirect or weak selection for ST. Second, the reproductive barrier between *xian*/*indica* and *geng*/*japonica* (hybrid sterility) has restricted gene flow, preventing the spread of *xian*/*indica*-derived tolerant alleles into *geng*/*japonica* gene pools [42,43]. Third, some ST-alleles, such as SKC1^184H^, may have originated relatively recently within the *xian*/*indica* lineage and have not yet had sufficient time or opportunity to introgression into *geng*/*japonica* [17,39]. Finally, the possibility of epistatic constraints cannot be ruled out: a ST-allele that is beneficial in a *xian*/*indica* background might be neutral or even detrimental in a *geng*/*japonica* background, thus being purged by selection [38,44]. Regardless of the exact causes, the observed rarity of most ST-alleles in *geng*/*japonica* strongly indicates that genetic improvement of ST in this subspecies will require targeted introgression of favorable alleles from *xian*/*indica* donors.

### 4.2. Subspecies-Specific Functional Comparisons of Three Representative SNPs Reveal the Critical Role of Genetic Background

In both *xian*/*indica* and *geng*/*japonica* populations, *RST1*^530A^ exhibits the strongest contribution to ST (confirmed by haplotype comparisons in *xian*/*indica*; 57.1% improvement in rSFW in *geng*/*japonica*) [17]. Notably, this conclusion directly corrects the earlier report that considered *RST1*^530G^ as tolerant and *RST1*^530A^ as sensitive [19]. Our *xian*/*indica* study already demonstrated through rigorous haplotype contrasts (Hap12 vs. Hap11) that *RST1*^530A^ is the tolerant allele [17]. The present study further validates this finding in a *geng*/*japonica* background: Hap7 varieties carrying *RST1*^530A^ (e.g., *Yanfeng47*, *Lindao20*) display significantly higher rSFW and lower DLR than that of Hap1 varieties under 150 mM NaCl treatment. More importantly, using chromosome segment substitution lines and the FBP-LUZ transcriptional reporter system, we provide the first mechanistic evidence in *geng*/*japonica*: the *RST1*^530A^ protein exhibits weaker repression of its target gene *OsAS1* expression compared to *RST1*^530G^, resulting in a higher upregulation of *OsAS1* under salt stress (Figure 5 and Figure 6). The reduced repressive activity of *RST1*^530A^ leads to increased *OsAS1* expression. This upregulation promotes asparagine synthesis, enhances nitrogen assimilation, reduces ammonium accumulation, and thereby improves ST [20]. Thus, the G530A substitution represents a partial loss-of-function for the *RST1* repressor protein, which translates into a gain-of-function for ST at the whole-plant level. *RST1*^530A^ is a functionally conserved, mechanistically well-defined core ST allele across subspecies, making it a priority target for cross-subspecies breeding. We note that the 57.1% contribution rate was estimated specifically in the Hap7 background; the consistency of the phenotype across all three Hap7 varieties supports its robustness, while the CSSL experiment provides independent validation in a near-isogenic context. Testing the effect of RST1^530A^ across diverse *geng*/*japonica* haplotypes would be a valuable future direction to assess the generalizability of this effect.

Collectively, three lines of evidence support the conclusion that the G530A substitution reduces *RST1* repressor activity at the protein level. First, in the CSSL experiment (Figure 5), *RST1* transcript levels are equivalent between HHZ and HHZ-CSSL6-JZ2, yet OsAS1 expression is significantly higher in the line carrying *RST1*^530A^, indicating a protein-level functional difference independent of transcript abundance. Second, in the FBP-LUZ assay (Figure 6), the two *RST1* protein variants exhibit differential repressive activity when expressed at equivalent levels from the same promoter, confirming that the G530A substitution directly alters the intrinsic biochemical activity of the *RST1* protein. Third, in the natural population (Figure 4), Hap7 varieties carrying *RST1*^530A^ show both lower *RST1* induction (1.94-fold vs. 2.60-fold) and higher *OsAS1* expression (6.97-fold vs. 4.55-fold) than Hap1 varieties, suggesting that both transcriptional and post-translational differences may contribute to the overall phenotype. Together, the CSSL and FBP-LUZ experiments establish that the G530A substitution itself directly reduces *RST1*’s repressor function, while the natural population data additionally suggest that transcript-level variation may contribute to the phenotypic difference between haplotypes.

A potential limitation of the FBP-LUZ assay is that it was performed in *N. benthamiana* leaves (dicot) rather than in rice (monocot) cells. For *RST1* to respond to salt stress in this heterologous system, the upstream signaling components—such as protein kinases that may phosphorylate and modulate *RST1* activity—must be sufficiently conserved between rice and tobacco. While this assumption is plausible given the broad conservation of core stress signaling pathways across higher plants, we acknowledge that the exact post-translational modifications of *RST1* and their regulatory dynamics may differ between species. Importantly, however, the primary purpose of the FBP-LUZ assay was not to recapitulate the entire native salt-signaling cascade, but rather to compare the intrinsic repressive activities of the two *RST1* protein variants (*RST1*^530A^ vs. *RST1*^530G^) under identical conditions. The observation that *RST1*^530A^ consistently exhibits weaker repression than *RST1*^530G^ when both are expressed at equivalent levels supports the conclusion that the G530A substitution directly alters the biochemical activity of the *RST1* protein itself. Future experiments using rice protoplasts or stable transgenic lines would be valuable to confirm these findings in a homologous system.

*SKC1*^140A^ contributes a 23.8–27.5% increase in rSFW in *xian*/*indica* [17] but only 16.5% in *geng*/*japonica* (Figure 3). Although significantly positive in both backgrounds, its effect is attenuated in *geng*/*japonica*. This may be attributed to the absence of the synergistic *SKC1*^184H^ allele in the *geng*/*japonica* gene pool. It is proposed that the two amino acid substitutions (P140A and R184H) lie on different sides of a cytoplasm-exposed loop of the *SKC1* protein and may exert additive effects on transport activity or phosphorylation status [39]. In *xian*/*indica*, ~31.6% of accessions carry both alleles and exhibit higher ST; in *geng*/*japonica*, *SKC1*^184H^ is entirely missing, so *SKC1*^140A^ acts alone and consequently less effectively. This again illustrates that ST is a polygenic trait, and the contribution of a single locus is conditioned by the presence of interacting partners in the genetic background.

The contrasting effect of *OsWRKY53*^173G^ between subspecies is striking: it confers a 38.6% improvement in rSFW in *xian*/*indica* [17] but shows no detectable benefit (and even a slight negative trend) in the *geng*/*japonica* population. One plausible explanation is that *OsWRKY53*’s regulatory function depends on the presence of specific interacting partners or target promoter contexts. In *xian*/*indica*, where *SKC1*^140A^ and/or *SKC1*^184H^ are present, *OsWRKY53*^173G^ may act in concert with other factors to enhance SKC1 expression. In *geng*/*japonica*, where these *SKC1*-tolerant alleles are largely absent (3.7% for *SKC1*^140A^, 0% for *SKC1*^184H^), this regulatory circuit may be non-functional. Alternative explanations include sequence variations in the *SKC1* promoter that alter *OsWRKY53* binding affinity, or differences in cofactor availability between subspecies. Distinguishing among these possibilities will require promoter-binding assays, bioinformatic analysis of WRKY-binding sites across the 215 varieties, or transgenic complementation in different *SKC1* backgrounds. Regardless of the underlying mechanism, the empirical observation that *OsWRKY53*^173G^ does not confer a detectable ST benefit in the 3H *geng*/*japonica* population has practical breeding implications: favorable alleles identified in *xian*/*indica* cannot be assumed to function equivalently in *geng*/*japonica* without population-specific validation.

### 4.3. Origin of RST1^530A^: Introgression from Xian/Indica into Geng/Japonica

Pedigree analysis and SNP linkage markers demonstrate that the *RST1*^530A^ allele in the *geng*/*japonica* varieties *Yanfeng47* and *Lindao20* is not endogenous to *geng*/*japonica* but was introgressed from the *xian*/*indica* variety *Aijiaonante* via a chromosomal segment. Several lines of evidence support this conclusion: (1) *Aijiaonante*, *Yanfeng47* and *Lindao20* share an identical combination of SNPs in the *RST1* coding region, including the key G → A transition at position +1743 bp (*RST1*^530A^). (2) SNP markers located 30 Kb upstream (*LOC_06g47100*) and 38 Kb downstream (*LOC_06g47220*) of *RST1* form the same haplotype in these three varieties, whereas the *geng*/*japonica* varieties *Lindao10* and *Nipponbare* belong to a different haplotype, indicating that at least a 68 Kb chromosomal segment was introgressed. This finding has important breeding implications [45]. First, it demonstrates that *xian*/*indica* rice is a valuable donor reservoir for *ST* genes in *geng*/*japonica*. Second, *Yanfeng47* and *Lindao20*, which have already acquired *RST1*^530A^ through conventional crossing, exhibit good ST and can serve as direct donor parents for transferring this allele into other elite *geng*/*japonica* varieties. Nevertheless, we acknowledge that our evaluations were conducted at the seedling stage under controlled hydroponic conditions. Field-based agronomic assessments across multiple seasons and locations will be necessary to fully validate the breeding potential of these varieties, particularly regarding yield-related traits and overall performance under natural saline field conditions, before they can be formally recommended as donor parents for large-scale breeding programs. Third, the KASP markers we developed can efficiently track the introgressed segment, facilitating marker-assisted selection.

To assess the potential contribution of neighboring genes, we surveyed the ~68 Kb region encompassing *RST1* (from *LOC_06g47100* to *LOC_06g47220*) using the rice reference genome annotation (MSU v7.0). Within this interval, in addition to *RST1* (*LOC_Os06g47140*/*Os06g0685700*), we identified several genes encoding proteins with known or putative stress-related functions, including *LOC_Os06g47100* (F-box domain-containing protein), *LOC_Os06g47160* (RING finger domain-containing protein), *LOC_Os06g47180* (WD40 repeat-containing protein), and *LOC_Os06g47210* (DNAJ domain-containing protein). The potential contribution of these neighboring genes to the salt-tolerance phenotype cannot be excluded. However, the *FBP-LUZ* assay provides independent biochemical evidence that the *RST1* protein itself exhibits allele-specific differences in transcriptional repression activity, supporting its role as a major contributor within this genomic segment. We acknowledge that high-density SNP arrays or whole-genome resequencing would provide a more precise delineation of the introgressed segment boundaries than the two-anchor-point approach used here. Such approaches are recommended for future studies aiming to fine map the introgressed tract.

Several limitations of this study should be acknowledged. First, we acknowledge that the nine SNPs surveyed in this study represent a targeted set of previously reported functional variants and do not capture the full allelic diversity at these seven loci; additional untyped variants within these genes may also contribute to ST. This limitation is inherent to a translational survey design and underscores the need for future fine-mapping or whole-genome resequencing efforts to comprehensively dissect the genetic architecture of ST in the 3H *geng*/*japonica* population. Second, the *RST1*^530A^ allele is rare in the 3H *geng*/*japonica* population (3.7%, 8/215), which limits the statistical power of haplotype-based comparisons. Nevertheless, the large effect size (57.1% relative increase in rSFW) and the consistent phenotype among all three Hap7 varieties support the biological robustness of this association. Third, the haplotype comparisons in natural populations may be influenced by linked variants or epistatic interactions, as the compared haplotypes differ at multiple SNP loci. We therefore interpret the contribution rates as empirical values rather than absolute genetic effects. Fourth, the HHZ-CSSL6-JZ2 line harbors a ~2.5 Mb introgressed fragment, which may contain additional genes that could contribute to the salt-tolerance phenotype; thus, the CSSL result alone cannot be interpreted as exclusive proof that *RST1* is the sole causal gene. However, our pedigree and SNP linkage analyses showed that a ≥68 Kb region encompassing *RST1* and its two immediate flanking genes co-segregates with the *RST1*^530A^ allele in the relevant varieties, although we acknowledge that this is a minimum estimate based on two flanking anchor points rather than a precisely defined introgressed tract. More importantly, the CSSL and *FBP-LUZ* experiments provide independent mechanistic evidence that is not subject to natural population sample size constraints or linked-gene confounding: (i) the CSSL analysis (Figure 5) compares near-isogenic lines on a uniform HHZ genetic background, with the introgressed segment carrying *RST1*^530G^ from *JZ2* substituted into the HHZ background. While this segment spans approximately 2.5 Mb and may contain additional linked genes that could contribute to the ST phenotype, (ii) the *FBP-LUZ* transcriptional reporter assay (Figure 6) provides independent biochemical evidence that the two *RST1* protein variants differ intrinsically in their repressive activity on the *OsAS1* promoter—an effect that is not subject to confounding by linked genes in the rice genome. The convergence of pedigree-based co-segregation of a ≥68 Kb core interval containing *RST1* with the 530A allele, near-isogenic CSSL phenotyping, and allele-specific biochemical reporter assays collectively supports the view that *RST1* is a major contributor—rather than the sole causal gene—to the ST phenotype associated with this genomic segment. Finally, all phenotypic evaluations were conducted at the seedling stage under controlled hydroponic conditions; field-based agronomic assessments are required to validate the breeding potential of the candidate varieties before large-scale utilization. Despite these limitations, the convergence of haplotype association, near-isogenic CSSL phenotyping, and biochemical reporter assays collectively support the view that *RST1* is a major contributor—rather than merely a linked marker—to the salt-tolerance phenotype associated with this genomic segment. We acknowledge that a direct gain-of-function experiment—introducing *RST1*^530A^ into a susceptible *geng*/*japonica* background—would be the ideal test to validate the breeding potential of this allele. Such experiments are planned as a priority for future work.

## 5. Conclusions

In this study, we investigated the distribution of nine ST associated SNPs from seven cloned genes in 215 *geng*/*japonica* varieties from the Huang–Huai–Hai region. Compared with our previous work in *xian*/*indica* rice, the *geng*/*japonica* population exhibits markedly lower frequencies of most tolerant alleles, with the exceptions of *OsHKT2;3*^77T^ and *RST1*^611G^. Functional assessment indicated that *OsSTL1*^289S^, *OsHKT1;1*^94K^, *RST1*^530A^ and *SKC1*^140A^ significantly improve ST in *geng*/*japonica*, among which RST1^530A^ showed the largest relative contribution (57.1% relative increase in rSFW). Notably, *OsWRKY53*^173G^ and *OsSTG5*^12S^ did not confer detectable benefits in the *geng*/*japonica* background, suggesting that their effects may depend on the presence of interacting SKC1-tolerant alleles. Mechanistically, our data suggest that *RST1*^530A^ enhances ST by more weakly repressing its target gene *OsAS1* compared with *RST1*^530G^. Pedigree and SNP linkage analyses further suggest that *RST1*^530A^ in the *geng*/*japonica* varieties *Yanfeng47* and *Lindao20* was likely introgressed from the *xian*/*indica* donor *Aijiaonante*. Collectively, this study provides a subspecies-specific evaluation of seven major ST genes, clarifies the functional role of *RST1*^530A^, and suggests *Yanfeng47* and *Lindao20* as promising candidate donor parents for the marker-assisted breeding of ST *geng*/*japonica* rice. While our molecular and seedling-stage evidence supports their potential, comprehensive field evaluations are required prior to large-scale breeding utilization.

## Data Availability

All data that support the findings in this study are available in this article and its Supplementary Files. The KASP genotyping data of 215 varieties are provided in Supplementary Table S1. KASP primers used for SNP genotyping are listed in Supplementary Table S2. The primers used for qRT-PCR are listed in Supplementary Table S3.

## Supplementary Materials

The following supporting information can be downloaded at: https://www.mdpi.com/article/10.3390/cimb48101011/s1.

## Abbreviations

The following abbreviations are used in this manuscript:

CSSL	Chromosome segment substitution line
DLR	Dead leaf rate
FBP	Fungal bioluminescence pathway
GWAS	Genome-wide association study
KASP	Kompetitive Allele-Specific PCR
qRT-PCR	Quantitative real-time PCR
rSFW	Relative shoot fresh weight
SNP	Single nucleotide polymorphism
ST	Salt tolerance
