# Supplementary material for: Dissection of Seven Saline Tolerance Genes in a Geng/Japonica Population Suggests the Subspecies-Specific Differentiation at RST1530A in Rice
Source: Curr Issues Mol Biol. 2026 Oct 1;48(10):1011. doi: 10.3390/cimb48101011 (PMC13703290; doi:10.3390/cimb48101011)
Supplement: Supplementary file 1 [file cimb-48-01011-s001.zip › Supplementary file 2.pdf]

Supplementary Table S2. KASP primers developed for SNP genotyping in this study.

SNP loci		Primer sequence (5′-3′)	Nucleotide type （S/T）[1]	Proportion of T type
	F1	GAAGGTGACCAAGTTCATGCTGCACACTGCCTCCGGCGAT		
STG5^{I12S}	F2	GAAGGTCGGAGTCAACGGATTGCACACTGCCTCCGGCGAG	**A/C**	**7.44%**
	R	GGAGGTGGGAGAACACGGTGT		
	F1	GAAGGTGACCAAGTTCATGCTCAGTGGCAAACCACCACCAC		
SKC1^{P140A}	F2	GAAGGTCGGAGTCAACGGATTCAGTGGCAAACCACCACCAG	**C/G**	**3.72%**
	R	CAGTGGCAAACCACCACCAG		
	F1	GAAGGTGACCAAGTTCATGCTTTAGGACGTCGGAACAAGCG		
SKC1^{R184H}	F2	GAAGGTCGGAGTCAACGGATTTTAGGACGTCGGAACAAGCA	**G/A**	**0**
	R	TTAGGACGTCGGAACAAGCA		
	F1	GAAGGTGACCAAGTTCATGCTGGCTTACCCTGGGGCTTGAG		
OsHKT1;1^{L94K}	F2	GAAGGTCGGAGTCAACGGATTGGCTTACCCTGGGGCTTGAA	**G/A**	**2.32%**
	R	GTAACCCTTTGGTAGTTCAGCTCGT		
	F1	GAAGGTGACCAAGTTCATGCTATGGTGGCTAATCCTATGACTA		
OsHKT2;3^{I77T}	F2	GAAGGTCGGAGTCAACGGATTATGGTGGCTAATCCTATGACTG	**T/C**	**99.53%**
	R	CACAAGTACAATCCTCGCTATG		
	F1	GAAGGTGACCAAGTTCATGCTGTCGAGCAAGAACGGCGC		
OsWRKY53^{A173G}	F2	GAAGGTCGGAGTCAACGGATTGTCGAGCAAGAACGGCGG	**C/G**	**6.98%**
	R	CCGTCCTCCAGCTTGTTGTTC		
	F1	GAAGGTGACCAAGTTCATGCTCTGAGACCTGCCCAGAGGG		
OsSTL1^{P289S}	F2	GAAGGTCGGAGTCAACGGATTCTGAGACCTGCCCAGAGGA	**G/A**	**14.42%**
	R	GAGCAAGGAGCTGGAAGCAG		
	F1	GAAGGTGACCAAGTTCATGCTCCCAGAACAACAAGAAATCAGATGG		
RST1^{G530A}	F2	GAAGGTCGGAGTCAACGGATTCCCAGAACAACAAGAAATCAGATGC	**G/C**	**3.72%**
	R	CCCAGAACAACAAGAAATCAGATGG		
	F1	GAAGGTGACCAAGTTCATGCTGCAGTGCCCTGTTTCCAGCG		
RST1^{E611G}	F2	GAAGGTCGGAGTCAACGGATTGCAGTGCCCTGTTTCCAGCC	**C/G**	**100%**
	R	CACAGATAATTTGTCATGTGGAGGAGT		

[1] S/T indicate the haplotype associated with saline sensitive (S) or tolerant (T), respectively. Underline represent the SNP sites.

Supplementary Table S3. Primers used for qRT-PCR in this study.

Gene ID	Foreard/Reverse	Primer sequence (5′-3′)
OsRST1	F	GTTGGTCCTATGTGCTATTTCCC
	R	AGACTCGACTGCAGTTTGTTAAG
OsAS1	F	TGGGTTATGAGGAAGGCGTT
	R	TTCAGGCCATCGATCCAGTT
OsACTIN	F	TGCTATGTACGTCGCCATCCAG
	R	AATGAGTAACCACGCTCCGTCA

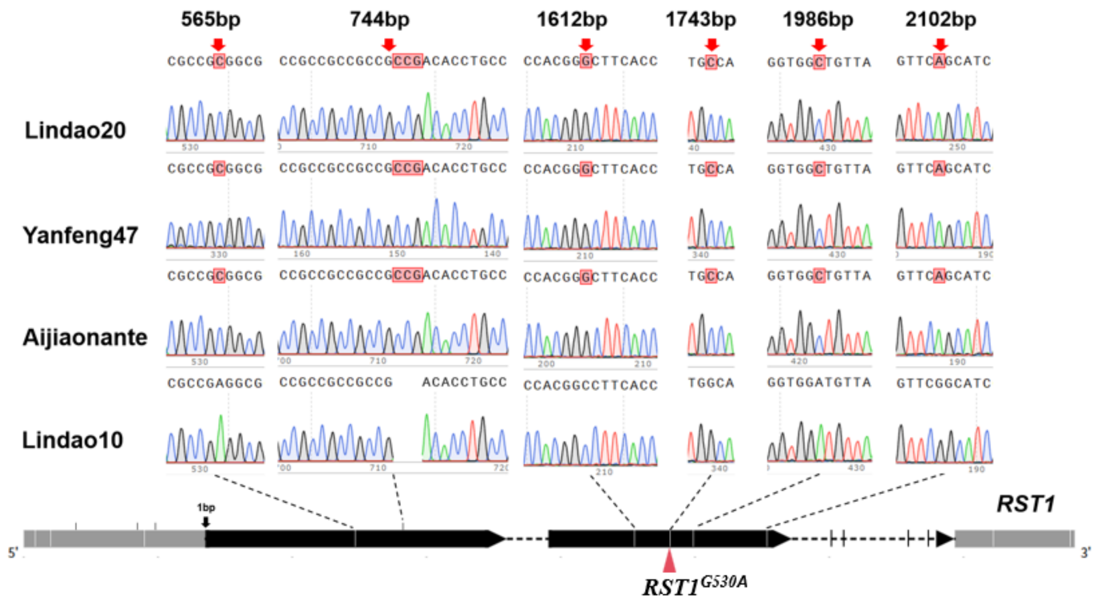

Supplementary Figure S1. Capillary sequence comparison of variations within the RST1 coding region between Lindao20, Yanfeng47, Aijiaonante and Lindao10.
